# *RB1*: a prototype tumor suppressor and an enigma

**DOI:** 10.1101/gad.282145.116

**Published:** 2016-07-01

**Authors:** Nicholas J. Dyson

**Affiliations:** Massachusetts General Hospital Cancer Center, Harvard Medical School, Charlestown, Massachusetts 02129, USA

**Keywords:** cell proliferation, E2F, tumor suppressor, pRB

## Abstract

The retinoblastoma susceptibility gene (*RB1*) was the first tumor suppressor gene to be molecularly defined. *RB1* mutations occur in almost all familial and sporadic forms of retinoblastoma, and this gene is mutated at variable frequencies in a variety of other human cancers. Because of its early discovery, the recessive nature of *RB1* mutations, and its frequency of inactivation, *RB1* is often described as a prototype for the class of tumor suppressor genes. Its gene product (pRB) regulates transcription and is a negative regulator of cell proliferation. Although these general features are well established, a precise description of pRB's mechanism of action has remained elusive. Indeed, in many regards, pRB remains an enigma. This review summarizes some recent developments in pRB research and focuses on progress toward answers for the three fundamental questions that sit at the heart of the pRB literature: What does pRB do? How does the inactivation of RB change the cell? How can our knowledge of RB function be exploited to provide better treatment for cancer patients?

## Abstract (precis)

In this review, Dyson summarizes some recent developments in pRB research and focuses on progress toward answers for the three fundamental questions that sit at the heart of the pRB literature: What does pRB do? How does the inactivation of RB change the cell? How can our knowledge of RB function be exploited to provide better treatment for cancer patients?

The textbook model for pRB function is appealingly simple (Fig. 1). pRB is a chromatin-associated protein that limits the transcription of cell cycle genes, primarily via regulation of the E2F transcription factor. In addition to binding to E2F, pRB interacts with chromatin regulators. These contacts allow pRB to recruit and stabilize complexes that repress transcription. By suppressing transcription of E2F targets, pRB restricts the expression of genes that are needed for cell proliferation. pRB is broadly expressed, but its activity is controlled by cyclin-dependent kinases (CDKs). Active pRB is found in quiescent cells, during G1 phase of the cell cycle, and during checkpoint-mediated cell cycle arrest. Hyperphosphorylation of pRB at the G1/S transition relieves pRB's inhibition of E2F and allows cell cycle progression. An extensive body of data shows that pRB is functionally compromised in many tumors either as a result of mutations in *RB1* or mutations that increase the phosphorylation of pRB or through the expression of viral oncoproteins that target pRB. The inactivation of pRB compromises the ability of cells to exit the cell cycle, and this places them in a state that is highly susceptible to oncogenic proliferation (for a review, see Hinds and Weinberg 1994; Weinberg 1995; Sherr 1996; Nevins 2001).

**Figure 1.**
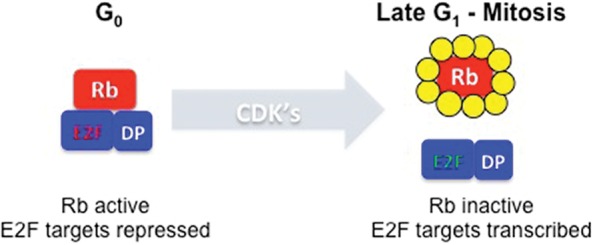
pRB and E2F provide cell cycle regulation of promoter activity. An interaction between pRB and E2F/DP heterodimeric complexes represses transcription of E2F-regulated promoters. This interaction can be detected in quiescent cells, differentiated cells, and cells arrested in G1 by activation of checkpoint pathways. When cells enter a cell division cycle, CDKs phosphorylate RB (depicted by yellow circles), leading to the disruption of E2F repressor complexes and the accumulation of activator E2F complexes that drive transcription.

As readers of the RB literature will appreciate, this description glosses over several inconvenient gaps in the data, and research over the past two decades has given us an increasingly complex picture of pRB action (Fig. 2). Although E2F is the best-known target of pRB, mapping the genome-wide distribution of pRB on chromatin has been technically challenging. Currently, there is surprisingly little information about precisely which genomic loci are controlled directly and specifically by pRB. The genomic distribution of pRB varies between cycling, quiescent, and senescent cells (Wells et al. 2003; Chicas et al. 2010; Ferrari et al. 2014; Kareta et al. 2015). It is uncertain what proportion of pRB is bound directly at E2F-regulated promoters or whether this is the most functionally relevant population of the protein, and it is unclear which or how many E2F-regulated promoters are truly rate-limiting for pRB-mediated control of cell proliferation.

**Figure 2.**
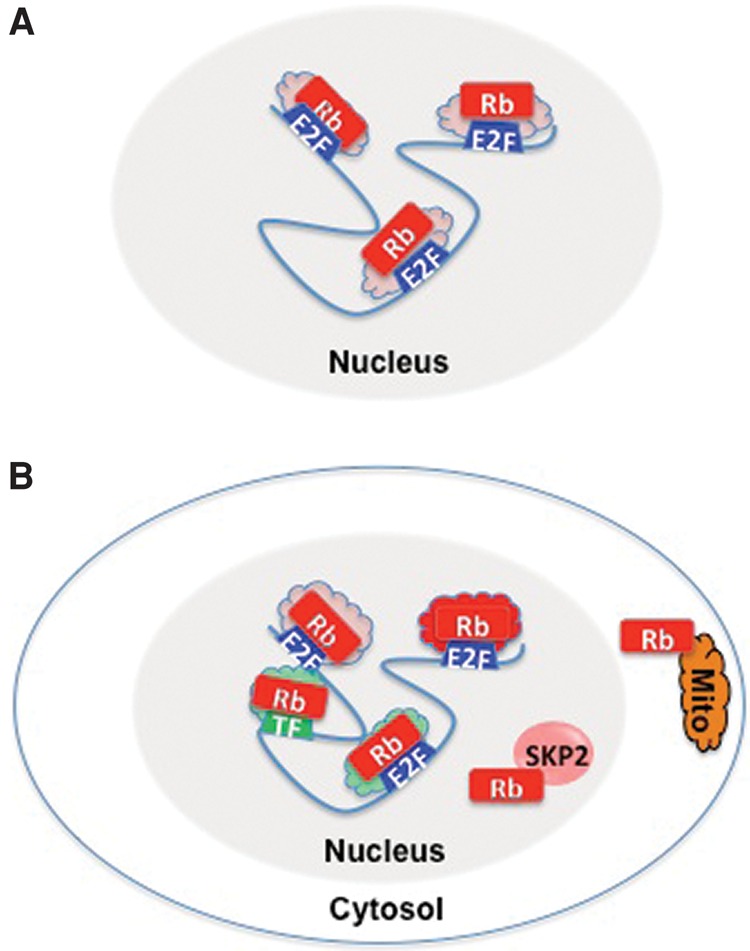
pRB has multiple mechanisms of action. (*A*) Shortly after the discovery of the interaction between RB and E2F, the model for pRB's mechanism action was relatively simple: pRB acts in the nucleus, where it associates with E2F complexes and represses promoters. Initially, the mechanism of repression was not known, and E2F targets were thought to be regulated in much the same way. (*B*) An updated model illustrating several of the layers of complexity that have been added to pRB's mechanism of action over the past two decades. Note that pRB recruits several different types of corepressors to E2F targets (depicted in red and pink), and, under certain conditions, E2F/RB complexes associate with coactivator complexes (green) and increase transcription of some targets. pRB does not act solely at E2F-binding sites but also associates with several transcription factors in addition to E2F. pRB has transcription-independent activities in the nucleus (illustrated here by its association with Skp2) and in the cytosol, where it associates with mitochondria.

In addition to the repression of E2F-regulated genes, pRB has been implicated in the organization of chromosomal domains and has roles in gene activation, particularly in response to apoptotic and differentiation signals (Thomas et al. 2001; Ianari et al. 2009; Calo et al. 2010). The RNA signatures associated with *RB1* mutation include both up-regulated and down-regulated transcripts (Black et al. 2003; Markey et al. 2007; Ertel et al. 2010). Multiple proteins have been implicated in pRB-mediated repression, but the mechanisms of pRB-mediated activation have not been characterized in as much detail. In addition to E2F, pRB associates at substoichiometric levels with a large number of nuclear proteins. Factors reported to interact with pRB include multiple cyclins, CDKs, and phosphatases (that act on pRB) as well as an assortment of chromatin-associated proteins that have varied activities (Harbour and Dean 2000; Morris and Dyson 2001; Talluri and Dick 2012). It is undoubtedly true that pRB is a cell cycle-dependent regulator of chromatin, but it is more accurate to describe pRB as a multifunctional, chromatin-associated protein rather than viewing it simply as a repressor of E2F.

pRB-mediated control of cell cycle progression also turned out to be more complex than initially imagined. pRB not only targets E2F but also has transcription-independent effects. Notably, a physical interaction with Skp2 enables pRB to regulate the stability of p27 (Ji et al. 2004; Binne et al. 2007). Indeed, in some assay systems, pRB-mediated cell cycle exit correlates better with its effects on p27 levels than with changes in proteins expressed from E2F-regulated genes (Ji et al. 2004). A pool of pRB has been detected at mitochondria, where it suppresses apoptosis (Ferecatu et al. 2009; Hilgendorf et al. 2013), providing further support for the view that pRB has effects on cell proliferation that extend beyond transcription.

RB function is especially relevant during tumorigenesis. Cancer genome sequencing confirmed that *RB1* is mutated in most retinoblastomas, osteosarcomas, and small-cell lung cancers, and it is mutated at lower frequencies in a variety of other cancer types. pRB is often described as a component of a regulatory pathway that is inactivated in most cancers (the INK4A/Cyclin D1/pRB/E2F pathway) (Kato et al. 1993; Aagaard et al. 1995; Koh et al. 1995; Lukas et al. 1995; Sherr 1996). The proteins in this “pathway” are actually components of a much larger network of cell cycle regulators. Significantly, individual types of cancer typically associate with particular lesions in this network (e.g., mutation of *RB1* in retinoblastoma, mutation of *INK4A* in pancreatic cancer, amplification of *Cyclin D1* in breast tumors, etc.). This selectivity suggests that the various perturbations of this “pathway” are not identical but that specific mutations have different consequences in different contexts. Data indicating that pRB retains some degree of E2F regulation in *INK4A* mutant cells or when phosphorylated by cyclin D-dependent kinases (Haberichter et al. 2007; Narasimha et al. 2014) and evidence that hyperphosphorylated pRB interacts with the mTORC2 complex and attenuates Akt activation (Zhang et al. 2016) support the view that the inactivation of pRB by phosphorylation is not functionally equivalent to the mutation of the *RB1* gene.

Animal studies demonstrate that the biological role of *RB1* is context-dependent. In much of the developing mouse embryo, pRB loss does not have major effects on tissue pathology. In specific compartments, the genetic ablation of *RB1* alters cell cycle progression/cell cycle exit, sensitivity to apoptosis, senescence, and differentiation (for review, see Vooijs and Berns 1999; Goodrich 2006; Viatour and Sage 2011; Sage 2012). The mutation of *RB1* can alter the type of differentiation programs that are activated, the extent of differentiation that occurs, and the ability of differentiated cells to permanently exit the cell cycle (for examples, see Thomas et al. 2001; Calo et al. 2010; for review, see Thomas et al. 2003; Sage 2012).

Viewed together, the many reported pRB-associated proteins and the evidence that the impact of *RB1* mutation is context-dependent highlight a central issue in RB research that is not fully resolved. On one hand, pRB's interaction with E2F has been conserved during evolution, and this role is evident in many different experimental systems. On the other hand, it is also clear that pRB has the ability to interact with various proteins, sometimes with context-specific effects. The relative importance of these two aspects of RB biology is uncertain. Does pRB primarily function in the same way in most cell types, with the variable effects of *RB1* inactivation mostly reflecting the impact of a complex phenotype in different situations, or is pRB such a multifunctional protein that its key mechanism of action is fundamentally different in different situations?

The textbook models of pRB function were built by combining results from different cell systems. In those early studies, it was generally assumed that results obtained in one cell type would be true in all others. In current research, much more emphasis is placed on understanding pRB's role in specific contexts. This thinking was influenced in part by evidence of a variable functional overlap between *RB1* and the two related genes p107 and p130 (Dyer and Bremner 2005) and also the observation that the expression of these three family members varies greatly during animal development (Jiang et al. 1997). The importance of context has been beautifully illustrated by the extensive efforts that have been devoted to identifying the precise cell type of origin of retinoblastoma (Chen et al. 2004; Dyer and Bremner 2005; Xu et al. 2009, 2014), an origin that differs between mouse models and the human disease. Such detailed studies were needed because of the possibility that there may be unique features to the molecular circuitry around pRB in these progenitor cells that could not be inferred by studying other cell types. Presumably, equivalent studies will be needed to understand the activity of pRB in each of the contexts in which it plays an important role. In a sense, RB research has matured from searching for a simple generic model that explains all observations to a more nuanced picture in which the precise role of pRB may vary and the consequences of *RB1* inactivation are extensive.

In summary, we know a great deal about what pRB can do. Despite the multitude of theories or perhaps because of the number of possibilities, it is difficult to pinpoint the precise mechanism by which pRB acts. As a result, pRB has remained an enigma—a tumor suppressor whose action is more easily described in general terms rather than in specific details. Despite this, there has been a great deal of progress. Below, I summarize some of the recent studies that provided new insights into the biochemical properties of pRB and studies that have examined the consequences of *RB1* inactivation. Ultimately, the most meaningful test of our understanding of pRB is whether this knowledge has been used to improve treatments for cancer patients, and the final section describes some of the progress toward the translation of this research.

## How large is the pRB interactome and how is it organized?

Shortly after the cloning of *RB1* (Friend et al. 1986; Fung et al. 1987; Lee et al. 1987), it was discovered that a set of viral oncoproteins directly targets pRB and that these physical interactions were necessary for the transforming properties of the viral products (DeCaprio et al. 1988; Whyte et al. 1988; Dyson et al. 1989). The notion that viral proteins might interfere with pRB's interaction with its normal cellular partners led to extensive searches for these pRB-interacting proteins. By 2001, >110 pRB-associated proteins had been reported, many of which bound to pRB in a manner that was disrupted by viral proteins or tumor-derived mutations (for review, see Morris and Dyson 2001). According to current interaction databases (Euorpoean Bioinformatics Institute [EBI]-IntAct, Molecular Interaction [MINT], Interologous Interaction Database [I2D], and String), there are >300 proteins that interact with pRB. These lists are useful starting points for discussion so long as one accepts that many of these interactions need additional validation.

In the absence of a quick, simple, and definitive assay for pRB's tumor suppressor function, there is little consensus on the number of “true” pRB partners, and it is uncertain how many of the reported interactions with pRB are functionally significant. In a few cases, mutant alleles of binding partners have been shown to modify the tumor phenotype associated with mutant *RB1* alleles (Yamasaki et al. 1998; Ziebold et al. 2003; Lasorella et al. 2005; Parisi et al. 2007; Wang et al. 2010; Sun et al. 2011). However, these alleles can have dominant effects, and it is unclear how much of the genetic interaction should be attributed specifically to the loss of the physical interaction between the proteins. Indeed, one of the most remarkable features of the pRB literature is that, even after close to 30 years of study, the molecular mechanism of pRB-mediated tumor suppression has not been definitively identified. There are no mutational studies of *RB1*, for example, showing that the precise elimination of pRB's interaction with a single partner (or even a class of proteins) eliminates its tumor suppressor activity. In the absence of definitive data, the mechanism of pRB-mediated tumor suppression remains a matter of debate. At present, it seems likely that pRB does not have a single activity but that it acts as a tumor suppressor through its effects on multiple targets.

The large body of literature on pRB-associated proteins may be evidence that pRB is a very versatile protein involved in many processes and capable of nucleating a variety of interactions (for review, see Dick and Rubin 2013). A contrarian viewpoint is that the length of these lists of interacting proteins is a testament to the sensitivity of molecular biology techniques but little more. An important but often overlooked feature of this literature is that different groups have focused on different partners of pRB, and very few of the reported interactions have been confirmed by independent studies. The vast number of potential interactions creates difficulties for structure/function studies on pRB; it is now extremely difficult to examine the potential impact of any *RB1* mutation on pRB's interactions with all of its possible partners. This task is compounded by the fact that most of the tumor-derived mutant alleles of *RB1* that have been characterized to date have extensive effects on protein structure or stability and are little use for separating activities (Dick 2007). As a result, each single study of pRB-associated proteins provides only part of the picture. For several years, it has been unclear how we should think about pRB's overall mechanism of action or how the different activities of pRB are controlled.

**Figure 3.**
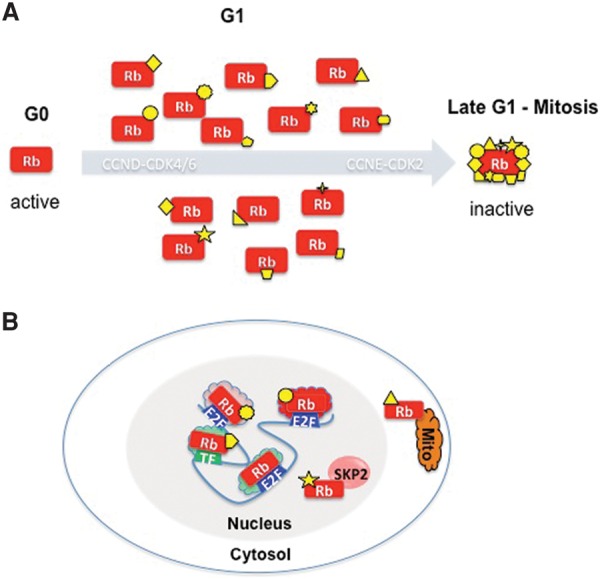
Multiple forms of pRB. (*A*) During G1 and in several types of arrested cells, pRB is hypophosphorylated. A recent study (Narasimha et al. 2014) revealed that this form of pRB is monophosphorylated on any one of 14 Cdk phosphorylation sites (denoted by the different yellow shapes) and converted to a fully inactive, hyperphosphorylated protein by Cyclin E/Cdk2 (or Cyclin A/Cdk2). Evidence that individual phosphorylation sites can selectively affect pRB's interaction with binding proteins leads to the speculation in *B* that specific monophosphorylation events may determine the localization and function of pRB. Note that the modified forms of pRB can coexist and that the post-translation regulatory code modulating pRB function need not be limited to phosphorylation but may also involve other types of protein modification. The specific functional properties of the monophosphorylated forms of pRB are as yet unknown.

Recent studies of RB structure have led to a new perspective on the pRB “interactome.” At 928 amino acids, pRB is a relatively large protein. It has not yet been possible to determine the structure of the full-length protein, but analyses of pRB fragments have provided valuable insights, and recent progress has shed light on the structural changes triggered by individual phosphorylation events (Rubin 2013). Remarkably, phosphorylation of T373 promotes a major conformational change that allows the N-terminal domain to dock against the pocket domain (Burke et al. 2010, 2012). Phosphorylation of S608 also triggers a conformational change in which a loop containing the phosphorylation site interacts with part of the pocket domain. In both cases, the structural changes driven by individual phosphorylation events alter specific binding domains but do not compromise the overall integrity of the protein and leave other binding surfaces intact. These observations are especially significant when combined with experiments that used isoelectric focusing gels to assess the number of phosphorylation events on endogenous pRB (Narasimha et al. 2014). Narasimha et al. (2014) reported that the “hypophosphorylated” form of pRB isolated from asynchronously dividing cells in tissue culture is entirely composed of monophosphorylated protein. Remarkably, the single phosphorylation event can be found at many, perhaps all, of the 14 known sites for CDK phosphorylation. Narasimha et al. (2014) show that monophosphorylated protein is the predominant form of pRB in contact-inhibited cells and cells arrested by DNA damage, situations in which pRB is known to be active (Fig. 3A).

These biochemical studies raise the fascinating possibility that there may be many different forms of “active” pRB in a cell. Indeed, a single cell may contain up to 14 different monophosphorylated forms of pRB, each potentially having a different set of binding partners. The idea that pRB's binding activity is tailored by phosphorylation is only part of the story. Evidence that pRB is both acetylated and methylated in specific contexts (Chan et al. 2001; Nguyen et al. 2004; Leduc et al. 2006; Munro et al. 2010; Saddic et al. 2010), evidence of interplay between different post-translational modifications (Carr et al. 2011; Macdonald and Dick 2012; Munro et al. 2012; Kim et al. 2015), and evidence that pRB interacts with specific partners in response to cellular cues (MacLellan et al. 2000; Miyake et al. 2000; Dick and Dyson 2003; Nguyen et al. 2004; Carr et al. 2014) all suggest additional levels of diversity. Collectively, these observations raise several intriguing possibilities: (1) There may be multiple pools of pRB that perform different functions. (2) Depending on their precise location (subcellular compartment or chromatin location) and modification, pRB molecules may interact with different sets of proteins. (3) The roles that pRB plays may be determined by signals that direct its specific post-translational modifications.

One of the appealing features of this model is that it helps to explain why pRB has been reported to interact with so many different proteins yet, at the same time, why so little of pRB is stably bound to any one of these partners. It also suggests how the multiple activities attributed to pRB might be regulated. If this model is correct, then pRB has a very complex mechanism of action (Fig. 3B). Clearly, a key goal for future studies of pRB will be to unravel this complexity: How many different types of pRB are there? How are these pools of pRB controlled? What are the biochemical properties of each different form of the protein? Which forms of pRB are key for specific molecular events and biological activities? The proof of the model will lie in the details and whether it is possible to define unique roles for specific isoforms of pRB.

## The cellular consequences of RB inactivation

Since it is the inactivation of pRB that is linked to tumorigenesis, understanding how cells change when *RB1* is mutated is a central issue. A clear picture of these changes may guide therapeutic strategies for targeting *RB1* mutant cells.

In agreement with the idea that pRB is a key negative regulator of proliferation, *RB1* loss has been demonstrated to lead to defects in cell cycle exit, facilitate entry into the cell division cycle, compromise G1/S arrest, and reduce senescence (for review, see Burkhart and Sage 2008; Sage 2012). The transcriptional signatures associated with *RB1* mutation include an up-regulation of many genes that are needed for cell proliferation, although careful examination shows that the mutation of *RB1* increases transcription of some, but not all, E2F targets (Hurford et al. 1997; Black et al. 2003). Recent analysis of transcriptional patterns in the mouse intestine shows that, in addition to E2F, Myc plays an important role in the altered transcriptional profiles of *RB1* mutant cells (Liu et al. 2015). Unexpectedly, *RB1* loss leads to the redistribution of both Myc and E2F3 proteins on chromatin, raising the possibility that these transcription factors act differently in *RB1* mutant cells compared with normal cells. Such observations underscore the fact that relatively little is known about the overall impact of pRB loss on chromatin biology.

A theme emerging from many different studies is the idea that that the functional consequences of *RB1* inactivation extend much further than the G1/S transition or the deregulation of E2F. One example of this is a series of studies showing that the loss of pRB affects progression through mitosis, increasing the incidence of lagging chromosomes and reducing the fidelity of chromosome segregation (Hernando et al. 2004; Iovino et al. 2006; Amato et al. 2009; Manning et al. 2010). These changes promote aneuploidy, particularly when combined with mutations in p53 (Zheng et al. 2002; Manning et al. 2014a). The mitotic phenotypes resulting from pRB loss have been linked to the altered expression of mitotic proteins (Hernando et al. 2004), reduced loading of the Condensin II protein CapD3 (Longworth et al. 2008; Coschi et al. 2010; Manning et al. 2010), reduced chromosomal cohesion (Manning et al. 2010; van Harn et al. 2010), and altered accumulation of cohesin complexes at pericentromeric chromatin (Manning et al. 2014b).

The mitotic defects associated with the inactivation of pRB are subtle. They do not lead to catastrophic mitotic failure, for example. At first glance, such changes might seem unimportant, particularly when compared with the abrupt cell cycle arrest seen when pRB is expressed in *RB1* mutant tumor cell lines such as Saos2 cells that are primed for arrest and senescence when they regain pRB (Hinds et al. 1992). However, chromosome instability and aneuploidy are common features of tumor cells. These phenotypes correlate with worse outcomes (Rajagopalan and Lengauer 2004; McClelland et al. 2009), and changes that increase chromosomal instability have been shown to promote resistance to targeted therapies (Sotillo et al. 2010). While retinoblastomas resemble other early-childhood cancers in having relatively low numbers of genetic lesions, pan-cancer studies show that mutations in the pRB pathway are associated with tumors that have elevated levels of gene copy number changes (Ciriello et al. 2013). Surveys of cell line and genomic data show that loss of one copy of *RB1* is associated with an increased level of genome instability (Coschi et al. 2014). Thus, the mitotic defects resulting from pRB inactivation may be relevant in many cancers. Significantly, the mitotic defects associated with pRB loss can be suppressed by knockdown of the checkpoint protein Mad2 (Hernando et al. 2004; Sotillo et al. 2010; Schvartzman et al. 2011), depletion of Wapl (to increase cohesin loading) (Manning et al. 2014b), addition of nucleosides (which improves replication dynamics and chromosome cohesion) (Bester et al. 2011; Burrell et al. 2013; Manning et al. 2014a), or manipulations that change chromatin marks at centromeric and pericentromeric heterochromatin (Manning et al. 2014b; Tanno et al. 2015). These raise the intriguing idea that it may be possible to reduce genome instability caused by *RB1* mutation.

Additional lines of evidence point to roles for pRB that seem separable from cell cycle control. A set of reports highlights a series of links between RB and metabolic pathways. *RB1* mutation (either alone or in conjunction with other pRB family members) causes change in metabolic pathways. These alterations include reduced mitochondrial respiration, reduced activity in the electron transport chain, changes in mitochondrial polarity, and altered flux from glucose or glutamine in *RB1* mutant cells (Sankaran et al. 2008; Clem and Chesney 2012; Nicolay et al. 2013, 2015; Reynolds et al. 2014; for review, see Nicolay and Dyson 2013; Lopez-Mejia and Fajas 2015). Indeed, proteomic studies show that changes in mitochondrial function are a major feature of *RB1* mutant mouse tissues (Nicolay et al. 2015). While the mechanistic basis for these metabolic changes has not been fully elucidated, these results are consistent with reports showing that E2F and RB proteins bind directly to promoters of genes encoding important regulators of metabolic flux, oxidative phosphorylation, and mitochondrial function (Cam et al. 2004; Hsieh et al. 2008; Chicas et al. 2010; Blanchet et al. 2011; Ambrus et al. 2013). Indeed, in *Drosophila*, E2F1 is needed for full activation of mitochondrial and muscle-specific genes during myogenic differentiation, and the presence of E2F in adult skeletal muscles is essential for animal viability (Zappia and Frolov 2016).

A key part of the explanation for the metabolic changes in pRB-deficient cells may stem from functional interplay between pRB, RBP2/KDM5a, and PGC-1α (PPARGc1A) (Varaljai et al. 2015). Varaljai et al. (2015) proposed that pRB promotes expression of these mitochondrial proteins by antagonizing a repressive activity of the KDM5a H3K4 lysine demethylase, thereby enhancing the effects of the PCG-1α coactivator. Remarkably, manipulations that increase mitochondrial function in pRB-deficient cells (inactivation of KDM5a and overexpression of PCG-1α) not only increase oxygen consumption rate but also suppress the muscle differentiation defects of *RB1* mutant cells. Changes in mitochondrial biogenesis have also been implicated in the ineffective erythropoiesis observed in *RB1* mutant mice (Sankaran et al. 2008), and other studies have shown that autophagy inhibitors also promote a healthy mitochondrial network in *RB1* mutant cells and promote differentiation (Ciavarra and Zacksenhaus 2010, 2011). Taken together, these studies suggest that metabolic changes are likely a major cause of the differentiation defects seen in *RB1* mutant mouse tissues (for review, see Benevolenskaya and Frolov 2015).

Other studies have shown that a subpopulation of pRB is located at the outer mitochondrial membrane, where it physically interacts with Bax and promotes apoptosis (Hilgendorf et al. 2013), suggesting that pRB loss impacts multiple aspects of mitochondrial function. This observation adds to a large number of studies showing that, under specific conditions, pRB can cooperate with other factors to promote the transcriptional activation of differentiation programs and regulate the expression of apoptotic regulators (for examples, see Thomas et al. 2001; Ianari et al. 2009; Calo et al. 2010; for review, see Attardi and Sage 2013).

In the light of such results, one might question whether the long-standing focus on the role of pRB/E2F in the control of the G1/S transition has given us a complete picture of the cellular changes that occur when *RB1* is mutated. Proteomic profiles of *RB1* mutant mouse tissues show that changes in the levels of proliferation proteins are not a uniform feature of *RB1* mutant tissues or the major proteomic effect of pRB loss (Nicolay et al. 2015). Substantial differences between the effects of pRB loss on mRNA and protein levels suggest that post-transcriptional controls are likely to play a significant role in determining the ultimate effects of *RB1* inactivation.

**Figure 4.**
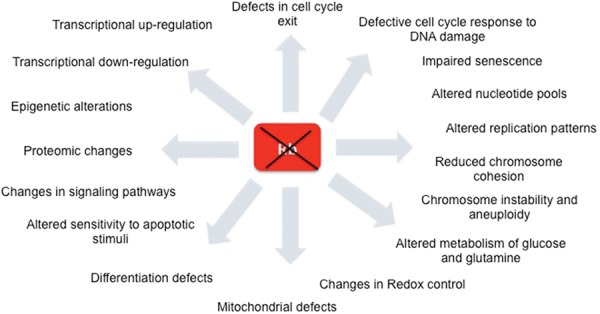
The consequences of *RB1* inactivation. The ablation of *RB1* impacts many cellular processes. These effects are highly interconnected, and it remains to be determined which specific changes are essential for tumorigenesis. A key goal in the immediate future will be to identify the consequences of *RB1* inactivation that can be best exploited therapeutically to target *RB1* mutant tumors.

Much more research is needed to understand how many cellular processes are altered when *RB1* is mutated. However, already it is clear that the consequences of *RB1* inactivation are far-reaching, affecting many aspects of cell biology (Fig. 4). Moving forward, the key questions will be: Which of these changes are relevant during tumorigenesis and which can be exploited to target cancer cells?

## The translation of RB research

The genetic lesions causing the frequent functional inactivation of pRB in tumors create two different types of challenges: In cells where *RB1* is mutated, the challenge is to identify features that distinguish *RB1* mutant cells from normal cells and represent points of vulnerability that can be exploited; in tumors where pRB is present but functionally inactivated, there is an additional possibility to reactivate the latent tumor-suppressive properties of pRB.

The development of effective inhibitors for Cdk4/6 kinases has been one of the most impactful applications of pRB research (Fry et al. 2004; for review, see Sherr et al. 2016). Complexes of Cdk4/6 and D-type Cyclins have multiple substrates, but it is clear that pRB is one of their most important targets. Cdk4/6 inhibitors have a capacity to activate pRB in G1 phase, and, in most normal cells, this triggers a reversible pRB-dependent cell cycle arrest. Cdk4/6 inhibitors also have pRB-independent effects, particularly when used at high concentrations. In contexts where deregulation of Cyclin D:Cdk4/6 kinases drives tumorigenesis, inhibition of these kinases can trigger cellular senescence or apoptosis (Fry et al. 2004; Thangavel et al. 2011; Choi et al. 2012; Sawai et al. 2012). Cdk4/6 inhibitors had modest effects when tested as a monotherapy in solid tumors (Flaherty et al. 2012; Dickson et al. 2013; Cadoo et al. 2014; DeMichele et al. 2015; Vaughn et al. 2015). This may reflect the fact that many signaling pathways converge on CDK regulation, and cells can express alternative CDKs that phosphorylate similar or overlapping sets of substrates. However, Cdk4/6 inhibitors have shown great efficacy when combined with other inhibitors targeting key mitogenic and/or survival pathways. Cdk4/6 inhibitors synergize strongly with inhibition of HER2, PI3K/mTOR, MEK, IGF1R/IR, and B-RAF (Finn et al. 2009; Franco et al. 2014; Heilmann et al. 2014; Vora et al. 2014; Yadav et al. 2014). In 2015, the Food and Drug Administration granted accelerated approval to a combination of the Cdk4/6 inhibitor palbociclib and letrozole for the treatment of hormone receptor-positive advanced breast cancer (Finn et al. 2015), and the efficacy of palbociclib in this setting has been confirmed in subsequent large-scale trials (Turner et al. 2015). Large-scale studies of mouse models of patient-derived xenografts show that Cdk4/6 inhibitors are synergistic with many different classes of compounds (Gao et al. 2015), and they may ultimately be useful in a variety of combination therapies.

Several strategies have been described for targeting *RB1* mutant tumor cells. The fact that *RB1* mutant cells fail to arrest at the G1/S transition in response to checkpoint signals (Harrington et al. 1998; Knudsen et al. 1998; Bosco et al. 2007) may explain why *RB1* mutant tumors are often sensitive to DNA-damaging agents and why some *RB1* mutant tumors initially respond well to treatment (Sharma et al. 2007; Ertel et al. 2010; Witkiewicz et al. 2012). The current models of RB function predict that the uncontrolled cell proliferation of *RB1* mutant cells is driven by deregulated E2F. Remarkably, studies using mouse models of retinoblastoma have shown that the short-term exposure of fetuses to E2F or CDK inhibitors is sufficient to suppress tumor formation in long-term assays (Sangwan et al. 2012). One might imagine that effective inhibitors of E2F activation would be high on the wish list of many pharmaceutical companies, but, to date, very little progress has been reported on this subject, and only a few compounds have been available for research (Ma et al. 2008; Sangwan et al. 2012; Kurtyka et al. 2014).

Unlike most *RB1* mutant tumors, retinoblastomas lack mutations in p53. These tumors develop from progenitor cells that are dependent on Mdm2 (Xu et al. 2009), and, as they also often express high levels of Mdm4 (Laurie et al. 2006), they may be targetable by agents such as Nutlin that activate p53 signaling (Elison et al. 2006; Laurie et al. 2006, 2007). Retinoblastoma tumor cells have also been reported to be selectively sensitive to inhibition of the Syk kinase (Zhang et al. 2012; Pritchard et al. 2014). However, follow-up studies were unable to show similar effects in orthotopic xenografts (Pritchard et al. 2014), and drug sensitivity profiles of a broad panel of cell lines do not show a general association between *RB1* status and sensitivity to Syk inhibitors (Garnett et al. 2012).

In most human cancers, *RB1* mutations occur in tumors that also mutate p53. Zhu and colleagues (Wang et al. 2010; Gordon et al. 2013; Zhao et al. 2013) have shown that inactivation of Skp2 can suppress proliferation of p53- and pRB-deficient cells in part through an up-regulation of p27 (Zhao et al. 2015) and the activation of E2F1-mediated apoptosis (Lu et al. 2014). This genetic interaction is effective at suppressing tumorigenesis in mouse models but has yet to be applied to human tumors. Based on genetic interactions that were discovered in *Drosophila*, others have suggested targeting TSC2 to elevate reactive oxygen species (ROS) in *RB1* mutant tumors (Li et al. 2010; Gordon et al. 2013). Potentially, other metabolic features of *RB1* mutant cells, such as the changes in mitochondrial activity, depletion of nucleotide pools, or changes in autophagy, might provide alternative therapeutic strategies (Angus et al. 2002; Tracy et al. 2007; Macleod 2008). Ultimately, there may not be a single vulnerability that is characteristic of all *RB1* mutant cells, but different strategies may be necessary for specific types of tumors. Changes that activate differentiation programs are one potential strategy (MacLellan et al. 2000; Lasorella et al. 2005; Lin et al. 2011). Recent work has suggested that Notch signaling may be important in mouse models of small-cell lung cancer, one of the most common types of *RB1* mutant cancers (George et al. 2015). Inactivation of *Sox2* has also been shown to suppress *RB1* mutant pituitary tumors in mouse models (Kareta et al. 2015). In part, these effects may reflect the context-specific signals that are important for the formation of tumor-initiating cells, signals that likely vary between cancer types. Collectively, these studies illustrate the fact that there may be many ways to target an *RB1* mutant cell, and there are good reasons to expect that the next decade will be a very exciting time for pRB research.

In summary, the retinoblastoma susceptibility gene was identified in the pregenomic era, at a time when the tools for molecular biology were relatively limited. Modern technologies are providing a wealth of new information, and, in keeping with this, the ideas about the role of pRB are evolving too. The notion that pRB suppresses E2F-regulated transcription is still true, but the emerging view is that pRB's molecular activity is far more complex than initially supposed, with many different forms of pRB and many potential partners. It is also evident that the mutation of *RB1* is not a surgical change that alters cell cycle control while leaving the cell otherwise unaffected. pRB loss causes extensive changes to chromatin organization, patterns of transcription, metabolic pathways, and the proteome. The idea that *RB1* mutation has such far-reaching effects is consistent with the fact that this change is sufficient, in some contexts, to cause cancer. However, the central issue in RB research has not changed at all: The challenge for us all is to use this information to improve cancer treatment.
